# The Effect of Sulfur and Nitrogen Doping on the Oxygen Reduction Performance of Graphene/Iron Oxide Electrocatalysts Prepared by Using Microwave-Assisted Synthesis

**DOI:** 10.3390/nano14070560

**Published:** 2024-03-22

**Authors:** Micaela Castellino, Adriano Sacco, Marco Fontana, Angelica Chiodoni, Candido Fabrizio Pirri, Nadia Garino

**Affiliations:** 1 Department of Applied Science and Technology—Politecnico di Torino, Corso Duca degli Abruzzi 24, 10129 Torino, Italy; fabrizio.pirri@polito.it (C.F.P.); nadia.garino@polito.it (N.G.); 2 Center for Sustainable Future Technologies @Polito—Istituto Italiano di Tecnologia, Via Livorno 60, 10144 Torino, Italy; adriano.sacco@iit.it (A.S.); angelica.chiodoni@iit.it (A.C.)

**Keywords:** doping, graphene, iron oxide, oxygen reduction reaction, microwave

## Abstract

The synthesis of novel catalysts for the oxygen reduction reaction, by means of a fast one-pot microwave-assisted procedure, is reported herein and deeply explained. In particular, the important role of doping atoms, like sulfur and nitrogen, in Fe_2_O_3_-reduced graphene oxide nanocomposites is described to address the modification of catalytic performance. The presence of dopants is confirmed by X-ray Photoelectron Spectroscopy analysis, while the integration of iron oxide nanoparticles, by means of decoration of the graphene structure, is corroborated by electron microscopy, which also confirms that there is no damage to the graphene sheets induced by the synthesis procedure. The electrochemical characterizations put in evidence the synergistic catalysis effects of dopant atoms with Fe_2_O_3_ and, in particular, the importance of sulfur introduction into the graphene lattice. Catalytic performance of as-prepared materials toward oxygen reduction shows values close to the Pt/C reference material, commonly used for fuel cell and metal–air battery applications.

## 1. Introduction

As society has progressed, energy consumption has rapidly increased in recent decades, and the development of alternatives to fossil fuels, such as renewable resources, represents today one of the most important goals for the scientific and industrial communities. Actually, this is considered a mandatory step to reduce the emission of greenhouse gases, a cause of climate change that drastically affects our planet. In the 2021 “Sixth Assessment Report”, the Intergovernmental Panel on Climate Change (IPCC) showed that in recent decades (1995–2014), the CO_2_ atmospheric concentration has reached the same values (360–400 ppm) that have been inferred for the Mid-Pliocene warm period (3.3–3.0 Ma). The main difference between these two periods is that our level of CO_2_ emissions is mainly due to human-related activities, while in the Paleolithic period, the emissions were ascribed only to environmental causes. To limit global warming below 1.5 °C, CO_2_ removal or mitigation have to be achieved, starting at least in 2030 [1]. So, in the vision of more sustainable and green energy, during the last few years, many attempts have been made to improve low-emission and high-efficiency technologies for energy storage and conversion, i.e., fuel cells and metal–air battery systems [2,3,4,5,6].

As it is well known, the limiting component for the large-scale commercialization of these devices is represented by the cathodic section, within which the oxygen reduction reaction (ORR) occurs. Due to the high kinetic barriers of the ORR, especially at room temperature, a catalyst is needed, and today, reference materials for this purpose are represented by noble metals supported by carbon (e.g., Pt/C). However, the high cost of such materials and their limited long-term stability drastically reduce the effective application of metal–air batteries and fuel cells. Therefore, developing alternative, low-cost, non-precious, highly active electrocatalysts represents, even now, an interesting challenge. In particular, promising substitutes of platinum are represented by transition metal oxides (TMOs) and doped carbon-based materials [7,8,9,10,11,12,13] that have already demonstrated good ORR performance, close to the reference Pt/C.

In the literature, many articles report how the introduction of heteroatoms (e.g., N, S, B, and P) into a carbon framework is able to effectively enhance the overall electrochemical activity for the ORR, as non-precious carbon-based catalysts [14,15,16,17,18]. Notably, co-doping has also been found to generally produce more active sites than single-atom doping, with a more efficient absorption capacity of oxygen molecules and, in the case of doping with S, leads to a better desorption of OH* [19]. One of the most promising doping combinations is actually represented by N- or S-doped carbon, in which the introduction of these moieties tends to induce carbon activation through spin density changes [20] that in turn affects the electronic structure, and by introducing a large number of active centers for oxygen adsorption, thus giving rise to synergistic effects in ORR catalysis [21,22,23]. Even though co-doped carbon materials theoretically possess promising properties, such as for energy storage applications [24], only few articles particularly regarding doped graphene report effective and simultaneous doping with sulfur and nitrogen [19,25,26,27,28].

On the other hand, decoration with non-noble metal oxides is widely considered a low-cost alternative to platinum-based catalysts. TMOs in fact represents a commercially affordable alternative due to their low price and high abundance, combined with good electrochemical performances [29,30]. In our case, the choice of Fe_2_O_3_ decoration has already been proven to guarantee additional functionalities on N-rGO surfaces [31] and, at the same time, to avoid cancerogenic elements like cobalt or nickel [32,33]. Other groups have already demonstrated the boost in ORR performance thanks to the addition of iron oxide clusters on commercial platinum-on-carbon electrocatalysts [34], as well as the synergist behavior of FeO_x_ nanoparticles added to the N-doped carbon matrix [35,36,37] thanks to the coupling of carbon-based nanomaterials’ excellent electronic conductivity and low charge transfer energy and the catalytic activity of metal oxide nanoparticles.

In this context, we present our work, in which we combine, for the first time (to the best of our knowledge), the co-doping of reduced graphene oxide (rGO) with both nitrogen and sulfur, with the additional catalytic activity of Fe_2_O_3_ nanoparticles, which creates a functionalized and doped high surface area structure showing remarkable electrochemical activity for the ORR compared to the bare rGO-based catalyst doped with nitrogen or sulfur. Furthermore, microwave-assisted synthesis allows us to control the size of nanoparticles, dramatically reducing the uncontrollable growth and agglomeration of iron oxide clusters, resulting in decreased catalytic behavior.

The microstructure of the as-prepared N-S-rGO/Fe_2_O_3_ nanocomposite was characterized by Transmission Electron Microscopy (TEM), which confirmed the presence of small nanoparticles of Fe_2_O_3_ and the homogeneous doping distribution in the carbon lattice due to N and S atoms. Meanwhile, thanks to X-ray Photoelectron Spectroscopy (XPS) analysis, all of the binding states and the precise chemical distribution of dopants were defined.

The electrochemical and catalytic properties were evaluated through different techniques, and it was demonstrated how, by introducing sulfur atoms into the graphene lattice in addition to nitrogen ones, it is possible to achieve effective improvement in the ORR efficiency of rGO-Fe_2_O_3_ nanocomposites, as already reported in our previous work [31]. Moreover, we were able to confirm how microwave synthesis can be an effective and interesting alternative to traditional hydrothermal synthesis. In fact, this procedure is low-cost, is green and energy-saving, and guarantees a good reduction of graphene oxide and homogeneous doping without damaging the graphene lattice. This happens in a very short time (10 to 20 min) compared to standard synthesis procedures that generally require hours or additional subsequent thermal treatments [38]. Therefore, the proposed material is a promising and interesting low-cost alternative to platinum-based catalysts, showing notable ORR activity thanks to the co-doping with sulfur and nitrogen, enhanced by the cooperation with iron oxide nanoparticles.

## 2. Materials and Methods

### 2.1. Catalyst Synthesis

All of the reagents were utilized as purchased without any subsequent purification. The co-doped rGO/iron oxide nanocomposite (named N-S-rGO/Fe_2_O_3_) was synthesized by means of the procedure reported hereafter. In a microwave reactor with a 100 mL Teflon vessel, provided with pressure and temperature probes (Milestone FlexyWave, Milestone Inc, Shelton, CT, USA), 50 mg of GO (Cheap Tubes Inc., Grafton, VT, USA) was added to 30 mL of DI water, with 20 mg of thiourea (Sigma-Aldrich, Milano, Italy). Then, 25 mg of FeSO_4_·7H_2_O (Sigma-Aldrich) was added and dissolved in the as-prepared mixture. Later on, the mixture with the precursors was subjected to ultrasonic vibrations (Elmasonic S, Elma Schmidbauer GmbH, Singen, Germany) for nearly 30 min and the resulting mixture was put under irradiation for 15 min at 180 °C (800 W maximum). The reactor was then cooled down to room temperature. The resultant suspension was then collected in small vessels, washed with DI water, and freeze-dried (Lio 5P, 5Pascal srl, Milan, Italy) to remove all of the water. A first reference (i.e., without iron oxide decoration) rGO-doped sample (named N-S-rGO) was also synthesized by following the same procedure without the addition of the iron precursor. A second reference sample (i.e., without doping atoms) made of bare rGO (named rGO) was also prepared by means of the same reported procedure for the aforementioned samples, but without the use of thiourea as a reducing and doping agent. The reduction was obtained thanks to the high temperature reached (180 °C) in the microwave vessel in an aqueous solution.

### 2.2. Physical and Electrochemical Characterization

TEM analysis was performed with a TALOS F200X instrument (Thermo Fisher, Waltham, MA, USA) equipped with quad-EDX (Energy Dispersive X-ray) detectors for elemental analysis and a Continuum S Spectrometer (Gatan) for Electron Energy Loss Spectroscopy (EELS). The analysis of the images and the EDX and EELS spectra was carried out with the Thermo Scientific Velox software (version 3.12) and Gatan Microscopy Suite (version 3.51). Concerning TEM sample preparation, the nanocomposite powder was suspended in pure ethanol and, after sonication in an ultrasonic bath, was drop-casted on a lacey carbon Cu TEM grid.

XPS measurements were carried out using a PHI 5000 Versaprobe X-ray Photoelectron Spectrometer (Physical Electronics, Chanhassen, MN, USA), equipped with a monochromatic Al K-alpha X-ray source (1486.6 eV energy) to obtain information related to the chemical composition of the materials. Survey and high-resolution (HR) spectra were acquired by using a spot size of 100 μm. Pass energies of 187.85 eV and 23.5 eV were chosen for the survey and HR spectra, respectively. A combined electron and Ar ion gun source was chosen to compensate for the charging effect that occurred during the measurements. Multipak 9.6 dedicated software was utilized for data treatment. All binding energies were referenced to the C1s peak at 284.5 eV. The background contribution in the HR spectra was subtracted by a Shirley function.

Raman analysis was performed by using a Renishaw InVia Reflex micro-Raman spectrometer, equipped with a cooled CCD camera, exploiting a 514.5 nm (green) laser excitation through a microscope objective (50×) in a backscattering light collection. Two cycles were performed for each acquisition to reduce the noise-to-signal ratio. The integration time was 10 s. A Si wafer reference was used to calibrate the detector. Background signal was subtracted by means of a linear function. The I_D_/I_G_ ratio was calculated by evaluating the height of the D and G peaks after background subtraction.

All electrochemical tests were performed at room temperature with a CHI760D electrochemical workstation and an ALS RRDE-3A rotating disk electrode apparatus. Catalyst samples were deposited on a glassy carbon disk/Pt ring working electrode (electrode area 0.1256 cm^2^) following the procedure reported in [10]. A Pt wire was used as a counter electrode and Ag/AgCl was used as a reference electrode. Unless otherwise stated, all measurements were performed in a 3-electrode configuration (disk/reference/counter electrodes) in a 0.1 M KOH oxygen-saturated aqueous electrolyte solution with a rotational speed of 2500 rpm. All potentials always relate to the reversible hydrogen electrode (RHE) and are calculated by the following formula:

(1) $$V_{RHE}=V_{Ag/AgCl}+0.059\;*\;pH+E_{Ag/AgCl}^{0}$$

where *V_RHE_* is the calculated potential with respect to RHE, *V_Ag/AgCl_* is the measured potential with respect to Ag/AgCl, *pH* refers to 0.1 M KOH (equal to 13), and *E*^0^*_Ag/AgCl_* is the Ag/AgCl/3.0 M KCl reference potential (equal to 0.21 V).

CV curves were acquired from 0.18 V to 1.18 V with a scan rate of 10 mV/s in an O_2-_ or N_2_-saturated electrolyte solution. RDE tests were performed using LSV in the potential range from 0.18 V to 1.18 V (cathodic scan) with a 5 mV/s scan rate and variable rotation speed in the range 625–2500 RPM. RRDE measurements were performed using LSV in a 4-electrode configuration (disc/ring/reference/counter electrodes) by screening the disk electrode from 0.18 V to 1.18 V (5 mV/s scan rate) and setting the ring potential to 1.18 V. Electrochemical Impedance Spectroscopy (EIS) measurements were performed at a fixed potential of 0.68 V, with an AC signal of 10 mV amplitude and a frequency range of 10^−2^–10^4^ Hz. Chronoamperometry curves were acquired at a fixed potential of 0.68 V. The results obtained were compared with a commercially available material, i.e., a Pt/C catalyst (20% by weight, Sigma-Aldrich), and with samples that did not contain the element sulfur, i.e., N-rGO and N-RGO/Fe_2_O_3_.

## 3. Results and Discussion

**Figure 1:**
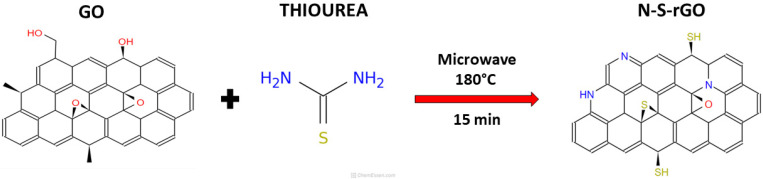
Schematic illustration of the synthesis of N-S-co-doped reduced graphene oxide.

In traditional hydro- or solvothermal reduction procedures of graphene oxide, high-temperature and high-pressure parameters are generally required for several hours [39]. Herein, the bare reference material (rGO and N-S-rGO) (see Figure 1) and Fe_2_O_3_-containing (N-S-rGO/Fe_2_O_3_) samples were prepared in few minutes by using the microwave-assisted method proposed without poisonous solvents or chemical precursors.

The functionalization with iron oxide nanoparticles together with the reduction and double-doping effect of the carbon lattice were confirmed by means of detailed chemical and morphological analysis. The goal was to demonstrate the effectiveness of the microwave-assisted synthetic procedure in introducing co-dopants in a fast, single-step process and to distinguish the real role of total sulfur functionalization on the catalytic performance of co-doped rGO.

### 3.1. Morphological Analysis

**Figure 2:**
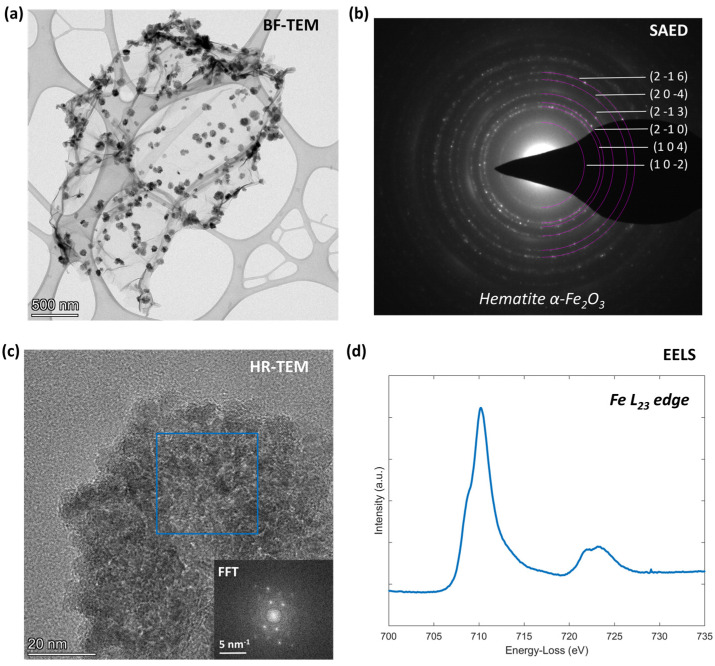
TEM analysis of the N-S-rGO/Fe_2_O_3_ sample: (**a**) Bright-Field (BF) TEM image of a single rGO flake decorated with Fe_2_O_3_ nanoparticles, (**b**) corresponding selected area electron diffraction (SAED) pattern, (**c**) high-resolution (HR) TEM image of an Fe_2_O_3_ nanoparticle with related Fast Fourier Transform of the highlighted region, and (**d**) Electron Energy Loss Spectroscopy (EELS) of the Fe L23 edge, acquired from a single nanoparticle.

Figure 2a provides a typical view of the morphology of the N-S-rGO/Fe_2_O_3_ nanocomposite powder, clearly showing the decoration of rGO flakes with iron oxide nanoparticles, with a characteristic lateral size of 58 ± 14 nm, as estimated from Bright-Field (BF) TEM images (such as Figure 2a). Although the majority of the analyzed rGO flakes show this kind of decoration, there are regions of the sample which are not completely decorated by the iron oxide nanoparticles (see Supplementary Materials for some examples). It is also important to notice that on the basis of morphological analysis, there is no evidence of induced damage on the rGO flakes due to the microwave-assisted synthesis of the iron oxide nanostructures. Based on electron diffraction data (Figure 2b), the nanoparticles exhibit a hematite (α-Fe_2_O_3_) crystalline structure (hexagonal unit cell, space group “R-3c”), in accordance with previously reported results with a similar synthetic approach [31] (see Supplementary Materials for a detailed description of the data analysis). High-resolution (HR) TEM images (Figure 2c) reveal the nanostructured external surface of the nanoparticles; inspection of the crystalline structure of the single nanoparticles through Fast Fourier Transform (FFT) confirms a regular crystalline arrangement corresponding to the α-Fe_2_O_3_ structure. The characteristic shape of the ELNES (Energy-Loss Near-Edge Structure) Fe L23 region acquired on a single particle (Figure 2d) confirms that the chemical environment of Fe atoms is compatible with the Fe_2_O_3_ phase [40,41,42].

**Figure 3:**
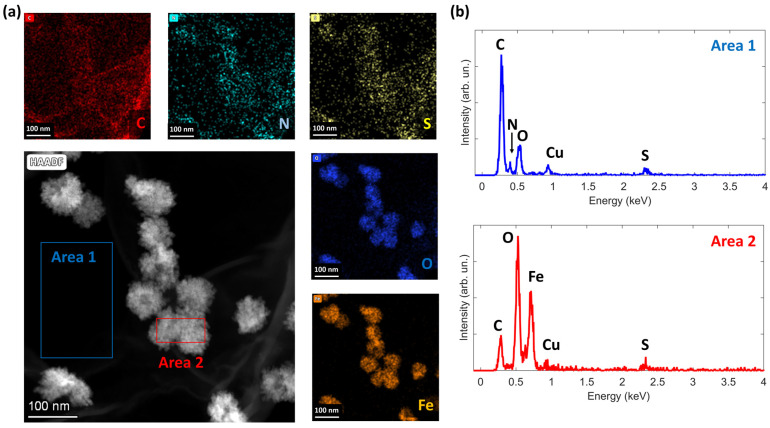
EDX analysis of the N-S-rGO/Fe_2_O_3_ sample: (**a**) High-Angle Annular Dark-Field (HAADF) Scanning Transmission Electron Microscopy (STEM) image, alongside corresponding EDX maps for C, N, S, O, and Fe elements; (**b**) sum EDX spectra obtained from Area 1 (bare rGO flake) and Area 2 (Fe_2_O_3_ nanoparticles).

Further spatially resolved elemental information is obtained by EDX imaging (Figure 3), which confirms two important facts: (1) the presence of Fe is confined to the nanoparticles and (2) the rGO flakes show co-doping with S and N atoms. Regarding point (2), the N and S signals are quite low, resulting in slightly noisy data in the EDX maps. However, their presence is definitely confirmed by the sum spectrum shown in Figure 3b (the Cu signal is due to the copper grid sample holder) and also by the XPS results described in the following section.

### 3.2. XPS and Raman Analysis

**Figure 4:**
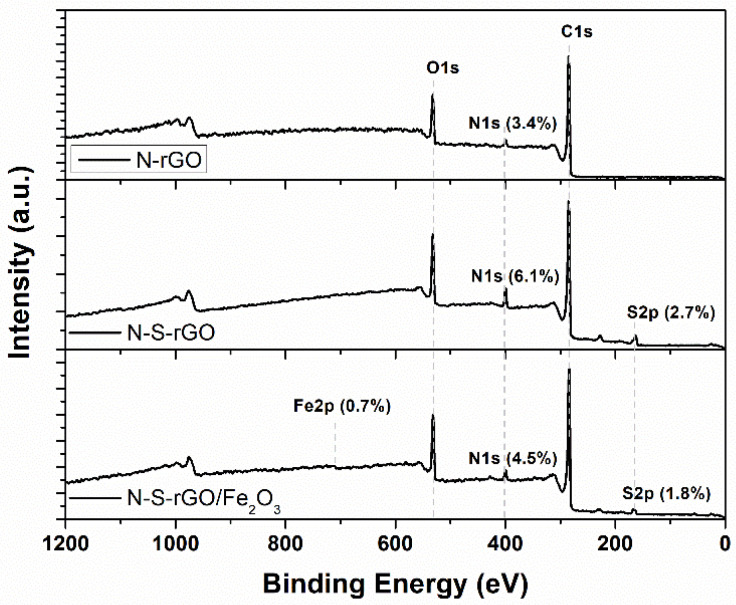
XPS survey spectra for N-rGO, N-S-rGO, and N-S-rGO/Fe_2_O_3_ samples (dopants’ relative atomic concentrations are reported together with Fe amount).

**Figure 5:**
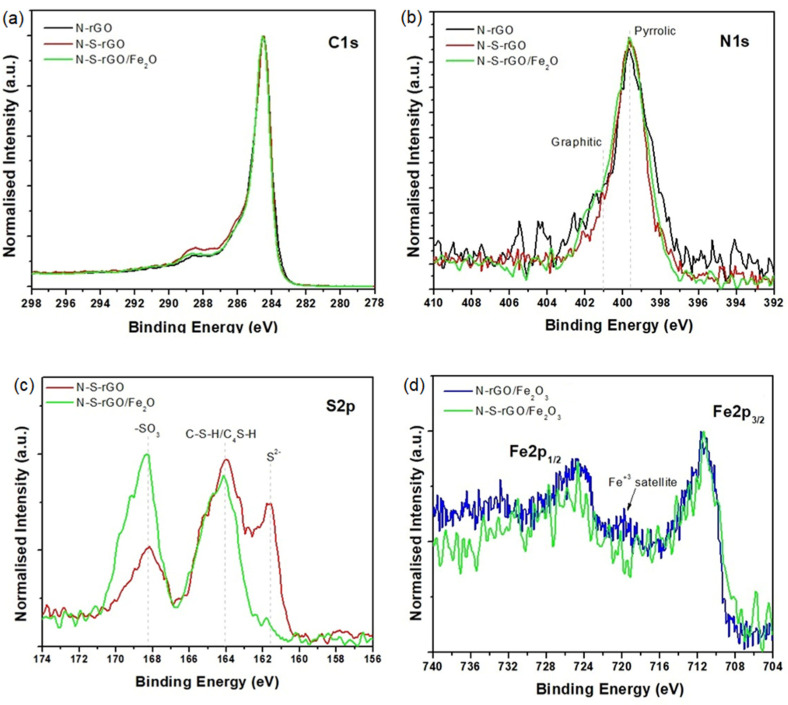
XPS HR spectra for (**a**) C 1s, (**b**) N 1s, (**c**) S 2p, and (**d**) Fe 2p photoelectronic peaks. For the sake of simplicity, in (**c**), the chemical shift has been reported only for the S 2p3/2 peak, while S 2p_1/2_ can be located 1.18 eV to the left of each S 2p_3/2_ peak. In (**d**), the blue curve refers to data published in Garino et al. [10].

XPS analysis has been performed on the N-S-rGO and N-S-rGO/Fe_2_O_3_ samples, together with the previously reported N-rGO one [5d], for comparison purposes. From the survey spectra (see Figure 4), we can infer the presence of C, O, and N in all of the three samples, S only in the thiourea-prepared samples, and Fe in the N-S-rGO/Fe_2_O_3_ samples. The relative atomic concentration (at.%) of the N 1s, S 2p, and Fe 2p peaks has been reported in the survey graphs. The highest amount of N and S has been detected for N-S-rGO, while N-rGO shows the lowest amount of N. The very low quantity of Fe detected in the N-S-rGO/Fe_2_O_3_ survey spectrum (0.7 at.%) compelled us to compare the HR spectrum, after having been acquired, to the Fe 2p HR spectrum already reported in [31] in order to establish the oxidation state thanks to a direct overlap between the two signals (see Figure 5d).

From the HR spectra, we can, first of all, check the rGO level of reduction induced by the synthesis procedure. The C 1s HR spectra (Figure 5a) show three perfectly overlapped curves, where there is only a small difference in the N-S-rGO sample, and in which the component due to the C=O bond (at 288.6 eV) and the one due to C-N/O/S (at 285.5 eV) are slightly higher than the other two. C-N and C-S bonds are located in the same chemical shift region (285.2–287.5 eV) [43]. Moreover, the lack of an evident difference between the curves suggests that the introduction of Fe at a low quantity into the rGO matrix does not create any new direct bonds between these chemical elements. If we move to the N 1s peak spectra (Figure 5b), we have again a perfect overlap between the curves (apart from the N-rGO sample, in which the signal is noisier than the latter two because it contains the lowest amount of N). Also, in this case, we cannot infer new bonds due to N-Fe atoms since the only chemical shifts detected are those due to pyrrolic and graphitic N in a C-based matrix [44]. A different story is told by the S 2p HR spectra (Figure 5c), in which we can see a big difference between the samples N-S-rGO and N-S-rGO/Fe_2_O_3_. In both curves, we can attribute the two different chemical shifts to thiol and/or thiophene (at 164.1 eV) and sulphonate (at 168.2 eV) [43], while the sample N-S-rGO shows a chemical shift due to sulfide or atomic S (at 161.5 eV) [45], which is not present in N-S-rGO/Fe_2_O_3_. Moreover, the relative concentrations due to -SH and C-SO_3_ groups are different in the two samples: 50.7% and 49.3% for N-S-rGO/Fe_2_O_3_, and 46.2% and 20.5% for N-S-rGO. This could be ascribed to the influence of sulphate groups present in the iron precursor. Periyasamy et al. [25] reported both thiophene and sulphonate groups in an N-S-co-doped rGO composite for ORR application, in which they attributed the catalytic activity only to the thiophene species, while the sulphonate groups were considered not active in the ORR process. The last verification has been conducted on the Fe 2p doublet by comparing the N-S-rGO/Fe_2_O_3_ and N-rGO/Fe_2_O_3_ curves, as already mentioned before. In Figure 5d, we can see that, apart from the noisy signal of the sample N-S-rGO/Fe_2_O_3_, the two curves show the same position for the Fe 2p_3/2_ (711.2 eV) peak and its related satellite, located at +8.5 eV on the binding energy scale. The relative peaks’ separation being equal to 8.5 eV is attributed in the literature to an Fe^3+^ oxidation state [46], in accordance with the results obtained by morphological characterization reported in the previous section. To further validate our hypothesis on N and S doping of the rGO matrix, we have also performed Raman analysis (see Supplementary Materials and Figure S4 for further details) on the bare rGO sample and the N-S-rGO one, finding typical spectra for a reduced graphene oxide structure, made up of G and D peaks [47]. According to B. P. Vinayan et al. [48], an increase in the I_D_/I_G_ ratio is expected with the inclusion of foreign atoms in the graphene lattice due to the increasing disorder in the crystalline structure. Accordingly, we have obtained I_D_/I_G_ = 0.84 for the rGO sample and I_D_/I_G_ = 0.91 for the N-S-rGO sample, respectively.

### 3.3. Electrochemical Analysis

The catalytic activity of the graphene-based samples was assessed through different electrochemical techniques, as reported here below (the N-rGO and N-rGO/Fe_2_O_3_ results were taken from our previous works) [10,31].

**Figure 6:**
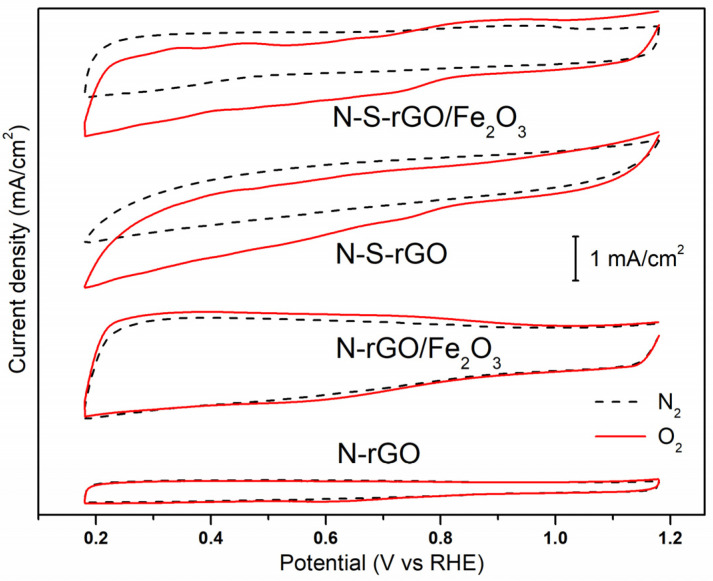
Cyclic voltammograms of different catalysts in O_2_-saturated and N_2_-saturated 0.1 M KOH solutions.

**Figure 7:**
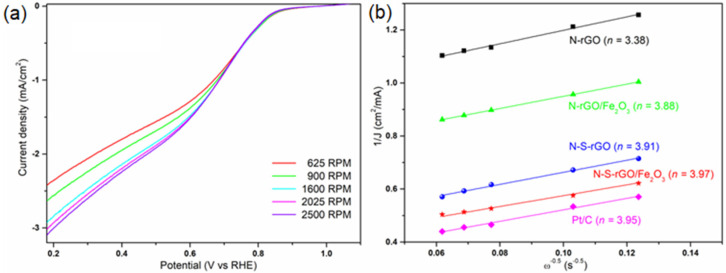
The results of the RDE measurements in a 0.1 M KOH solution: (**a**) ORR polarization curves of the N-S-rGO/Fe_2_O_3_ sample at different rotation speeds; (**b**) Koutecky–Levich plots of the different catalysts at a 0.38 V potential (the numbers between parentheses represent the calculated electron transfer number values).

Figure 6 shows the Cyclic Voltammetry (CV) curves acquired in both N_2_- and O_2_-saturated 0.1 M KOH electrolytic solutions. All of the samples exhibit a reduction peak in the cathodic scan at potentials in the range 0.6–0.8 V. This peak demonstrates the capability of the rGO-based catalysts to carry out the ORR, since the peak is not present in the nitrogen-related curve. Moreover, it is interesting to notice that the presence of sulfur atoms has a positive effect on the graphene samples, since the onset potential is shifted toward higher values with respect to bare nitrogen-doped catalysts. The same effect has been already observed by Bag et al. [19]. The catalytic pathways of the ORR, namely, the direct reduction of molecular oxygen to hydroxide ions (exploiting four electrons) and the indirect reduction to peroxide ions (exploiting two electrons) [49], were investigated through rotating disk electrode (RDE) measurements. The Linear Sweep Voltammetry (LSV) curves of the N-S-rGO/Fe_2_O_3_ sample, acquired at different rotation speeds ω, are shown in Figure 7a. Similar behaviors have been obtained for the rest of the samples. In agreement with a diffusion-controlled reaction [10], the reduction current increases with the increasing rotation rate. By fixing a potential value, the Koutecky–Levich equation (Equation (2)) can be applied to the data in Figure 7a to extrapolate the number of electrons n involved in the reaction [50]:

(2) $$\frac{1}{J}=\frac{1}{0.62nFC_{O_{2}}D_{O_{2}}^{2/3}\upsilon^{-1/6}\omega^{1/2}}+\frac{1}{J_{K}}$$

where *J* and *J_K_* are the measured and the kinetic current densities, respectively, *F* is the Faraday constant, *C_O_*_2_ and *D_O_*_2_ are the oxygen bulk concentration and diffusion coefficient, respectively, and *υ* is the kinematic viscosity of the electrolyte.

In Figure 7b the Koutecky–Levich plots of the different catalysts are compared. As previously investigated [31], the bare N-rGO sample is characterized by a mixed two-and-four-electron reduction pathway, while the functionalization of this structure with hematite nanoparticles shifts the reaction toward the four-electron path. The co-doping of graphene with sulfur and nitrogen further enhances the catalytic properties, leading to an n value as high as 3.97 for the N-S-rGO/Fe_2_O_3_ sample, which is comparable to the Pt-based one. In addition, it has to be highlighted that the N-S-rGO sample is characterized by better performance with respect to other nitrogen and sulfur co-doped graphene reported in the literature [19,39,51,52] and summarized in Table S2 of the Supplementary Materials.

**Figure 8:**
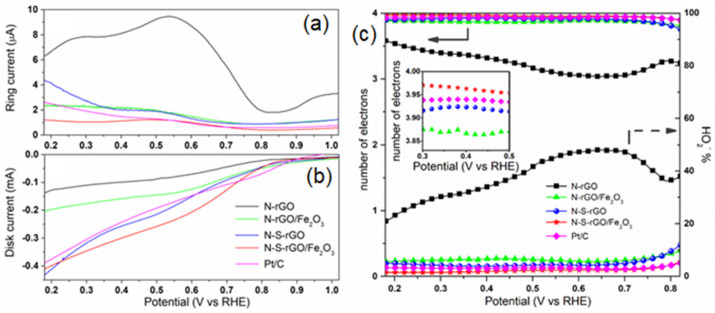
Results of RRDE measurements in 0.1 M KOH solution: (**a**) ring and (**b**) disk currents measured at 2500 RPM rotation speed; (**c**) comparison of electron transfer number (left axis) and peroxide percentage (right axis) calculated from curves in (**a**,**b**).

Rotating ring disk electrode (RRDE) measurements were then performed in a 0.1 M KOH solution to study the dependence of the electron transfer number on the applied potential and to compute the extent of the peroxide production. To this aim, the ring current IR, associated with a two-electron pathway, and the disk current ID, associated with a four-electron pathway, are reported in Figure 8a,b, respectively. Except for the N-rGO sample, the ID/IR ratio is larger than 100 in the whole potential range, thus demonstrating that for these catalysts, the direct reduction toward hydroxide is preferred [10]. On the contrary, for the bare N-doped sample, the ratio is about 10, meaning that a non-negligible reduction current is destined for the production of peroxide ions. These outcomes are quantified by Equations (3) and (4) reported below:

(3) $$n=4\times \frac{I_{D}}{I_{D}+I_{R}/N}$$

(4) $${{HO}^{-}}_{2}\%=200\times \frac{I_{R}/N}{I_{D}+I_{R}/N}$$

where *HO*^−^_2_% is the percentage of produced peroxide species and *N* is the current collection efficiency of the Pt ring [53].

**Figure 9:**
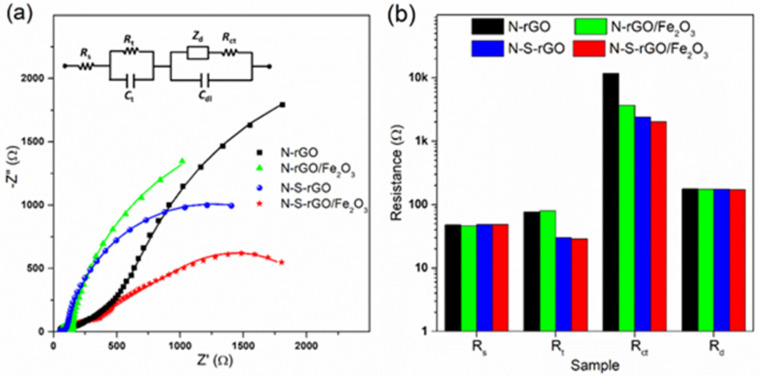
The results of the EIS measurements on different samples in a 0.1 M KOH solution: (**a**) Nyquist plots acquired at a 2500 RPM rotation speed and a 0.68 V potential (the points represent the experimental data, while the continuous lines are calculated from a fitting procedure using the equivalent circuit shown in the inset); (**b**) calculated resistances.

The results are summarized in Figure 8c. N-rGO can produce HO^−^_2_ ions with percentages as large as 48% at 0.65 V, and even if this value decreases for lower potentials, it never reaches as low as 20%. As a consequence, n values between 3 and 3.5 were obtained for this sample, demonstrating a mixed two-and-four-electron pathway. On the other hand, decoration with the iron oxide and/or the sulfur doping shifts the catalytic pathway toward the four-electron route; as evident in the inset of Figure 8c, the performance of the N-S-rGO/Fe_2_O_3_ sample resembles that of the reference Pt catalysts, with peroxide production lower than 5%. This result demonstrates that the synergetic effect of S and N co-doping and of Fe_2_O_3_ functionalization can boost the performance of graphene-based catalysts when compared to bare co-doped or hematite-decorated materials previously reported in the literature [9b, e] [39,54,55]. Three components can contribute to the total activity of a catalyst material, namely, the electric conductivity, the capability to efficiently exchange charges with the oxygen, and the efficient diffusion of ionic species [56]. With each of these processes, an equivalent resistance can be associated: the transport resistance R_t_, the charge transfer resistance R_ct,_ and the diffusion resistance R_d_, respectively. The extent of these resistances for the different catalysts can be obtained by Electrochemical Impedance Spectroscopy (EIS) analysis [57]. The Nyquist plot for all of the samples is shown in Figure 9a, in which the experimental data are reported together with the curves simulated, with the equivalent circuit shown in the inset; in the latter, a series resistance Rs is present to account for the electrolyte conductivity.

The obtained values for the different resistances are summarized in Figure 9b. The series resistances are equal for all of the samples, since the employed electrolyte is the same. Also, the diffusion resistances are comparable for the different catalysts, calculated by the Warburg impedance given below [57]:

(5) $$Z_{d}=R_{d}\sqrt{\frac{\omega_{d}}{j\omega }}tanh\sqrt{\frac{j\omega }{\omega_{d}}}$$

where *ω_d_* is the diffusion characteristic frequency. This can be explained considering that the morphology of various nanocomposites is analogous, and thus, the charge can diffuse in a similar way. Concerning the transport resistance, a large difference is present among the N-doped and the N-S-co-doped samples; this increase in the electric conductivity of graphene-based structures has already been observed after co-doping with metal atoms and nitrogen, with respect to bare nitrogen-doped catalysts [44]. Finally, it can be inferred that sulfur doping is also effective in reducing the charge transfer resistance, facilitating the exchange with molecular oxygen thanks to the thiol functionalities [58]. Moreover, the hematite nanoparticles can further decrease the resistance, making N-S-rGO/Fe_2_O_3_ the best-performing sample.

**Figure 10:**
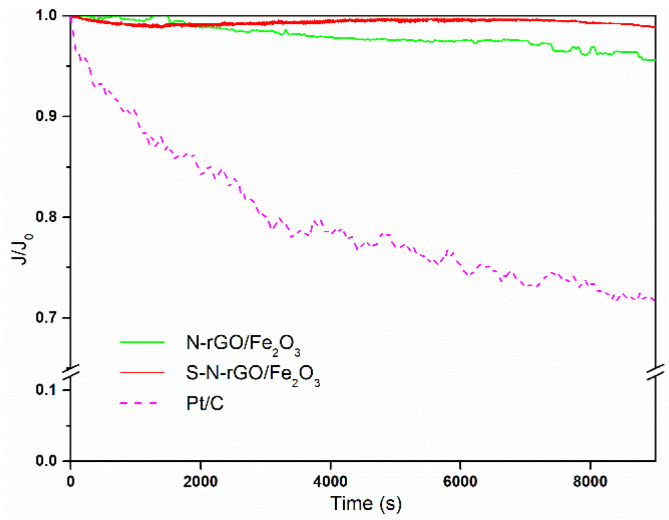
Chronoamperometric curves measured at 0.68 V potential and 2500 RPM rotation speed in 0.1 M KOH solution normalized with respect to initial current value.

The optimal performance of the S-N-rGO/Fe_2_O_3_ nanocomposite catalyst is also reflective of its excellent durability, as shown in Figure 10. After 9000 s, the current exhibits a reduction of only 1.5% with respect to the initial value. This result is in accordance with TEM analysis after chronoamperometry, which shows no significant changes in the morphology of the catalyst (see Supplementary Materials, Figure S3). In the same period, N-rGO/Fe_2_O_3_ is characterized by a decrease of 5%, while Pt/C exhibits a decline of 29%. When compared to other S-N-co-doped graphene [26], our composite material exhibits improved long-term stability, once again reinforcing that the simultaneous presence of the co-doping and the hematite decoration synergistically renders S-N-rGO/Fe_2_O_3_ an effective catalyst for the ORR.

## 4. Conclusions

In this work, we propose an efficient nanocomposite as an ORR catalyst based on rGO, presenting co-doping with nitrogen and sulfur atoms and an additional functionalization with iron oxide nanoparticles, obtained by using a fast and green microwave-assisted process. Morphological and structural characterization confirmed the integration of sulfur and nitrogen atoms in the graphene lattice and the decoration of Fe_2_O_3_ nanocrystals on its surface without damaging the rGO matrix during the synthesis procedure. The bare sample (without nanoparticles) and the nanocomposite were investigated as catalysts for the ORR. A comparison with our previous work on similar catalysts puts in evidence how the introduction of sulfur atoms into the graphene lattice, as a second dopant, gives rise to a considerable improvement in the electrochemical and catalytic properties. This is due to the introduction of additional active centers which enable better absorption of oxygen molecules and their consequent reduction, without any competition with the catalytic behavior of iron oxide nanoparticles dispersed on the rGO layers. The as-obtained co-doped nanocomposite catalyst is characterized by improved conductivity and higher charge transfer properties, in comparison to single-doped and/or non-decorated graphene. The proposed material shows excellent behavior for the ORR, with a performance competitive with that of commercial reference Pt/C catalysts and a well-improved stability with respect to the same reference catalysts.

## 5. Patents

A National Italian Patent (number 102018000010540) was obtained in 2020 (30 October) for the rGO production method here reported, titled “Reduced and doped graphene oxide, and its production method” (“*Ossido di graphene ridotto e drogato, e suo metodo di produzione*”).

## Data Availability

Data presented in this study are available on request from the corresponding authors.

## Supplementary Materials

The following supporting information can be downloaded at: https://www.mdpi.com/article/10.3390/nano14070560/s1, Figure S1: Bright-Field TEM images (a,b) and High-Angle Annular Dark-Field STEM images (c,d) of four different rGO flakes of the N-S-rGO/Fe_2_O_3_ sample; Figure S2: Selected area diffraction patterns corresponding to two different regions in the sample N-S-rGO/Fe_2_O_3_; Figure S3: TEM analysis of sample N-S-rGO/Fe_2_O_3_ after chronoamperometry for 5 h. (a–c): Bright-Field images; (d) representative EDX spectrum obtained for a region of interest. F contribution is due to Nafion, K and Ca contribution is from the aqueous solution, and Cu is from the TEM grid; Figure S4: Raman spectra of bare rGO and N-S-rGO samples; Table S1: Electron diffraction results obtained from two different regions (SAED 1 and SAED 2); the corresponding inter-planar spacing ratios were calculated by converting the radius of the diffraction rings (expressed in pixels) into 1 nm units using calibrated camera constant values; Table S2: Comparison of ORR properties of N-S-co-doped graphene-based electrocatalysts (all of the potentials refer to RHE). Eonset: onset potential; E1/2: half-wave potential; Jk: kinetic current density; GF: graphene framework; PDA: polydopamine; CN: carbon nanosheets. References [10,19,25,26,28,39,48,52,59] are cited in the Supplementary Materials.
