# Supplementary material for: The Effect of Sulfur and Nitrogen Doping on the Oxygen Reduction Performance of Graphene/Iron Oxide Electrocatalysts Prepared by Using Microwave-Assisted Synthesis
Source: Nanomaterials (Basel). 2024 Mar 22;14(7):560. doi: 10.3390/nano14070560 (PMC11013293; doi:10.3390/nano14070560)
Supplement: Supplementary file 1 [file nanomaterials-14-00560-s001.zip › nanomaterials-2914864-supplementary.pdf]

Supporting Information

The Effect of Sulfur and Nitrogen Doping on the Oxygen Reduction Performance of Graphene/Iron Oxide Electrocatalysts Prepared via Microwave-Assisted Synthesis

Micaela Castellino*, Adriano Sacco, Marco Fontana*, Angelica Chiodoni, Candido F. Pirri, and Nadia Garino

Table of contents

Supporting TEM analysis	3
TEM analysis of tested samples	6
Comparison with the published literature	8
Raman analysis	8
References	9

SUPPORTING TEM ANALYSIS

Additional morphological TEM analysis is provided in Figure S1, showing low-magnification images of different regions. It is interesting to notice that the decoration with Fe_2O_3 nanoparticles is not perfectly homogeneous: some regions show a higher density of nanoparticles (Figure S1-a vs. Figure S1-c), while some flakes are not decorated with nanoparticles (Figure S1-b and d).

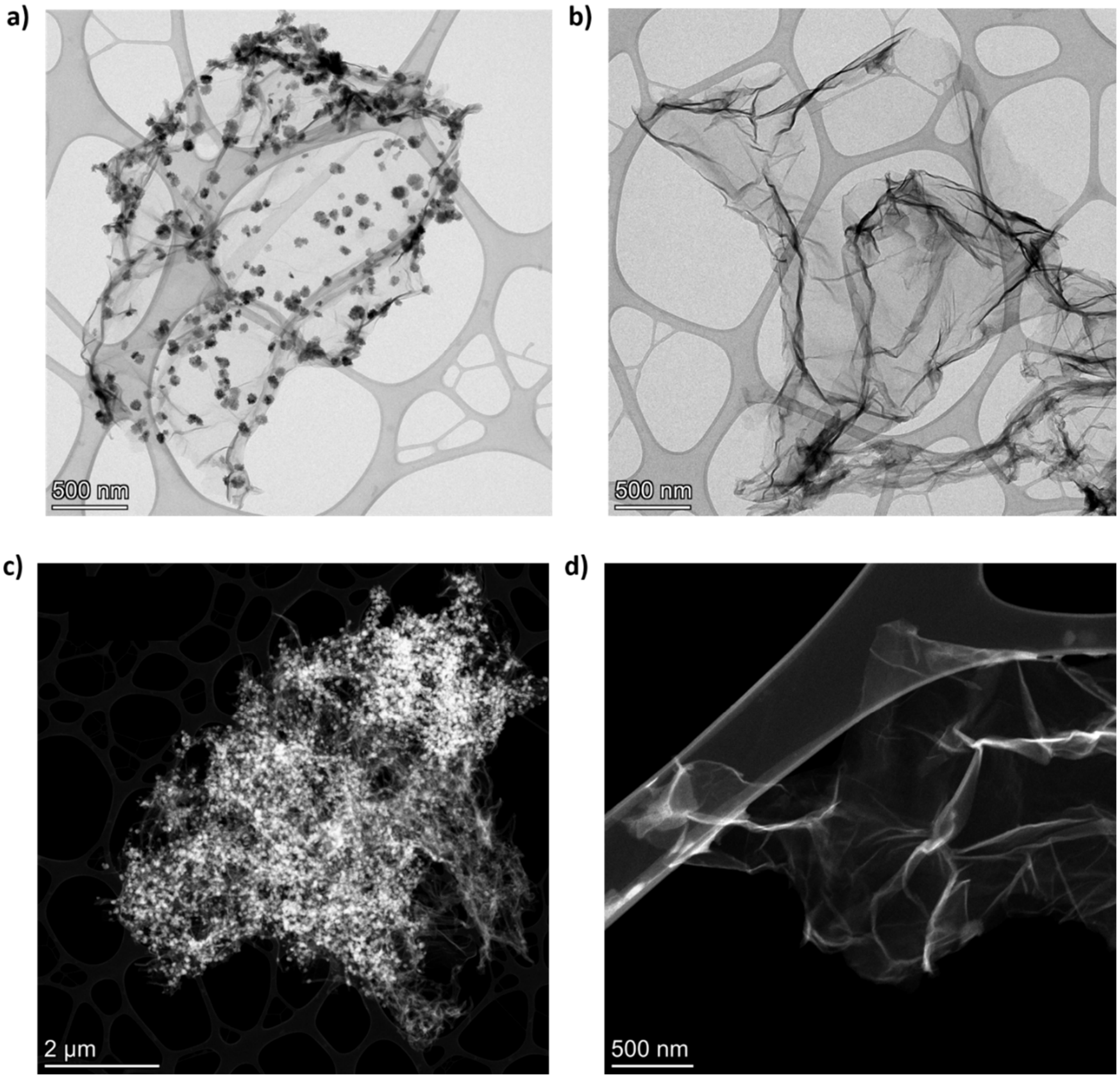

Figure S1: Bright-Field TEM images (a-b) and High-Angle Annular Dark-Field STEM images (c-d) of four different rGO flakes of the N-S-rGO/Fe_2O_3 sample.

Analysis of Selected Area Electron Diffraction patterns was carried out with Gatan Microscopy Suite software using the Circular Hough analysis script. As a reference for the hematite α-Fe_2O_3 crystalline structure, data reported in the American Mineralogist Crystal Structure Database (code: 0000143) were used (hexagonal unit cell, space group “R-3c”, a = b = 5.038 Å, c = 13.772 Å). Table S1 reports a comparison between inter-planar “d” spacing ratios in the reference structure and the ratios calculated from the electron diffraction patterns obtained from two different regions (SAED 1 corresponding to Figure 2-b in the manuscript and SAED 2), confirming that the nanoparticles exhibit a hematite structure.

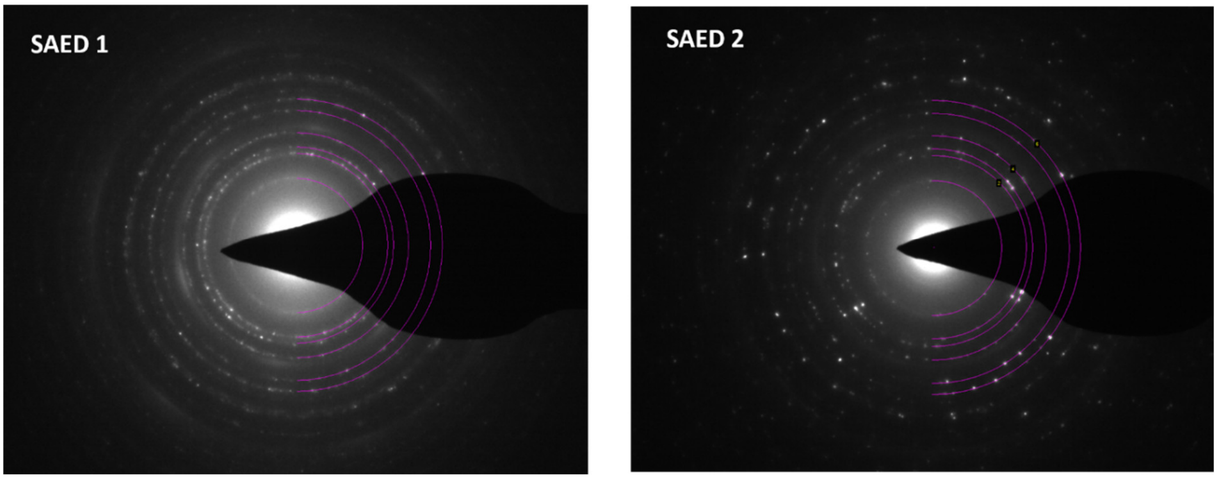

Figure S2: Selected area diffraction patterns corresponding to two different regions in the sample N-S-rGO/Fe_2O_3.

Table S1: Electron diffraction results obtained from two different regions (SAED 1 and SAED 2); the corresponding inter-planar spacing ratios were calculated by converting the radius of the diffraction rings (expressed in pixels) into nm^{-1} units using calibrated camera constant values.

REF Hematite	Planes (hkl)	d (Å)	d/d (104)	SAED 1	Ring	Rad (pixel)	d/d (ring 2)
	(10-2)	3.686	1.36		1	306	1.36
	(104)	2.703	1.00		2	417	1.00
	(2-10)	2.519	0.93		3	446	0.93
	(2-13)	2.208	0.82		4	511	0.82
	(20-4)	1.843	0.68		5	612	0.68
	(2-16)	1.697	0.63		6	663	0.63
				SAED 2	Ring	Rad (pixel)	d/d (ring 2)
					1	307	1.36
					2	416	1.00
					3	449	0.93
					4	509	0.82
					5	613	0.68
					6	667	0.62

TEM ANALYSIS OF TESTED SAMPLES

After 5 h of chronoamperometry testing at 0.68 V, the N-S-rGO/Fe_2O_3 catalyst was analyzed by means of electron microscopy. Figure S3 shows the results obtained from Bright-Field imaging and EDX analysis. Concerning sample preparation, the electrode was immersed in pure ethanol and sonicated for 5 minutes in order to detach the catalyst. In this way, a dispersion was obtained which could be drop-casted onto a lacey carbon Cu grid. As a consequence of the TEM preparation approach, the sample shows contamination from Nafion, which is part of the electrode preparation process for the electrochemical characterization.

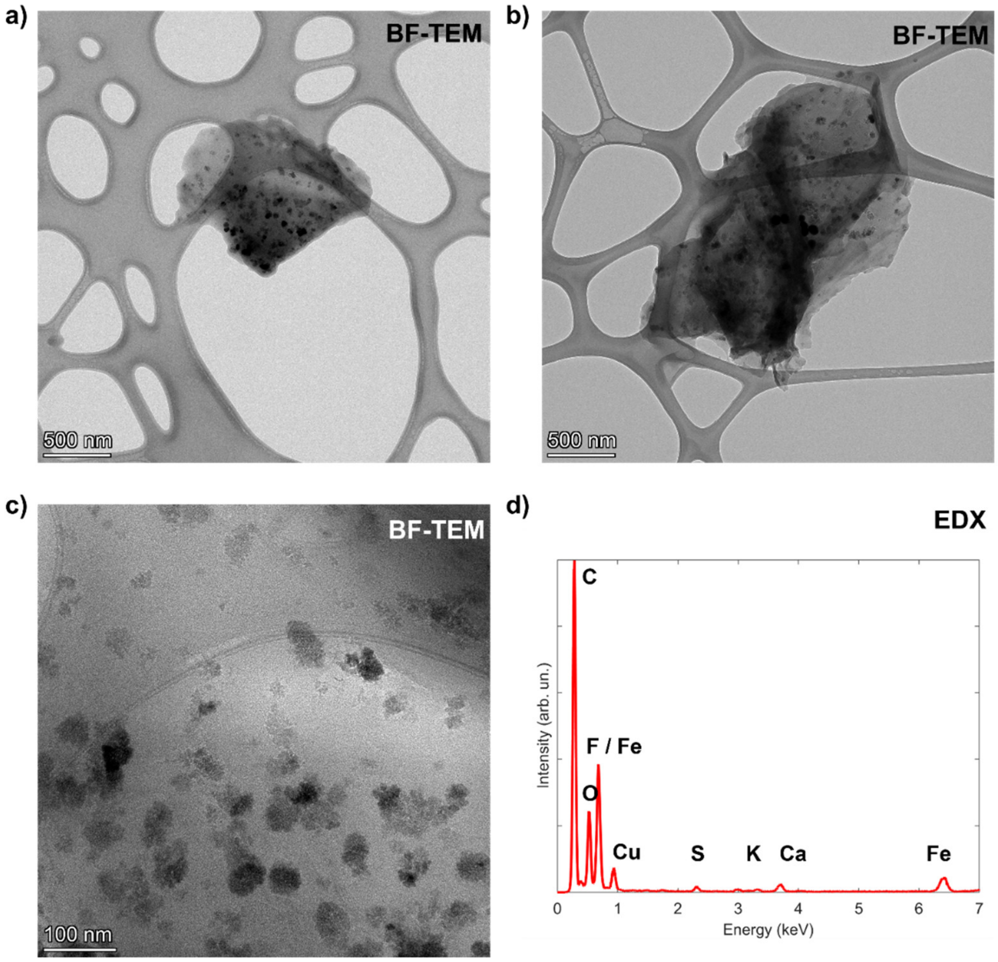

Figure S3: TEM analysis of sample N-S-rGO/Fe_2O_3 after chronoamperometry for 5 h. a), b), and c): Bright-Field images; d) representative EDX spectrum obtained for a region of interest. F contribution is due to Nafion, K and Ca contribution is from the aqueous solution, and Cu is from the TEM grid.

From the low-magnification images (Figure S3 a-b), it is clear that the characteristic morphology of the N-S-rGO/Fe_2O_3 sample is retained: iron oxide nanoparticles (confirmed by EDX, Figure S3-d)

decorate the rGO flakes. The characteristic size of the iron oxide particles (evaluated in images such as Figure S3 c) is (50 ± 12) nm, which is comparable with the value before testing, (58 ± 14) nm, as reported in the main manuscript. TEM analysis suggests that the N-S-rGO/Fe_2O_3 does not undergo significant changes, in accordance with the excellent stability reported in the manuscript.

COMPARISON WITH THE PUBLISHED LITERATURE

Table S2: Comparison of ORR properties of N-S-co-doped graphene-based electrocatalysts (all of the potentials refer to RHE). E_{onset}: onset potential; $E_{1/2}$: half-wave potential; J_k: kinetic current density; GF: graphene framework; PDA: polydopamine; CN: carbon nanosheets.

Material	Synthesis route	E_{onset} (V)	$E_{1/2}$ (V)	J_k (mA/cm^2)	n	Reference
SN-rGO	single-step non-hydrothermal chemical approach	0.916	--	7.7	3.50	[19]
3D N/S-GFs	one-pot hydrothermal approach	0.757	--	3.9	3.90	[26]
N/S co-doped graphene	self-polymerization of PDA+ reaction with cysteine	0.933	--	--	3.36	[52]
SN-rGO	mechanical grinding + pyrolysis method	0.850	--	--	3.80	[25]
N,S-CN	pyrolysis of GO-PDA hybrids	0.927	0.777	16.0	3.98	[28]
NS-G	hydrothermal approach + pyrolysis method	0.950	0.840	--	3.86	[39]
NS-G	solid-phase pyrolysis method	0.875	0.768	--	3.80	[59]
N-S-rGO/Fe_2O_3	MW-assisted hydrothermal approach	0.885	--	2.4	3.97	This work

RAMAN ANALYSIS

Additional vibrational spectroscopy analysis is provided in Figure S4 by means of Raman analysis. The measurements have been performed on a bare reduced graphene oxide sample (rGO), obtained by means of microwave reduction, as described in the manuscript in the *Catalyst Synthesis* section, and a co-doped rGO (N-S-rGO) sample, prepared by means of microwave-assisted hydrothermal synthesis with thiourea, to observe differences in the structure due to the inclusion of N and S atoms

in the graphene-like matrix. According to a previous study [48], in which B. P. Vinayan et al. demonstrated a change in the Raman spectra between bare rGO and S-rGO samples, we expected the same behavior in the I_D/I_G ratio, ascribable to both N and S atoms' inclusion by doping. In fact, the ratio increased from $I_D/I_G = 0.84$ (rGO) to $I_D/I_G = 0.91$ (N-S-rGO). There was also a weak shift toward lower Raman shift values, observed by comparing G and D peaks for the rGO sample (G = 1589 cm^{-1}; D = 1351 cm^{-1}) and the N-S-rGO one (G = 1583 cm^{-1}; D = 1344 cm^{-1}), in accordance with [48]. Thus, as already demonstrated for the N-rGO sample reported in our previous work [10], the doping, attributed to N and S atoms, induces a change in the graphene lattice by increasing its structural distortion.

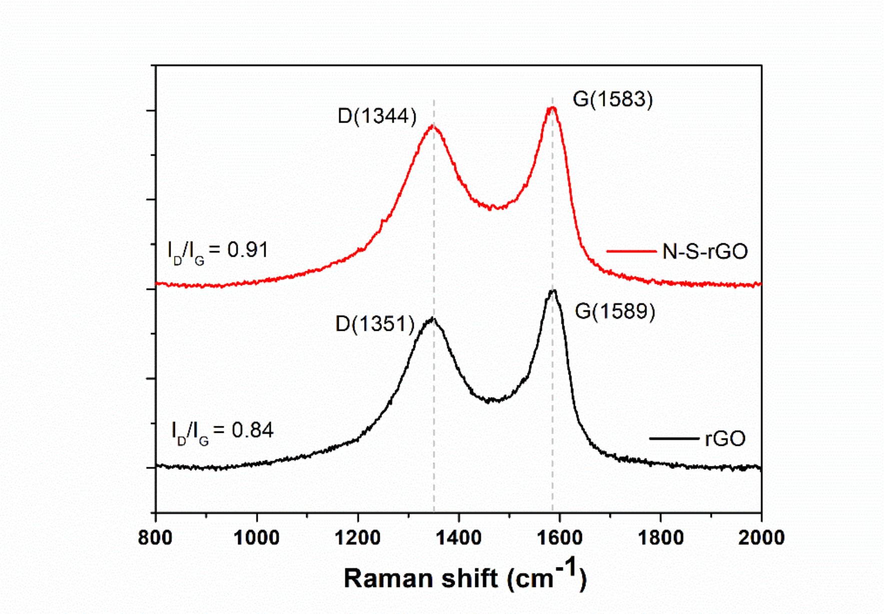

Figure S4 : Raman spectra of bare rGO and N-S-rGO samples.

REFERENCES

[10] N. Garino; A. Sacco; M. Castellino; J. A. Muñoz-Tabares; A. Chiodoni; V. Agostino; V. Margaria; M. Gerosa; G. Massaglia; M. Quaglio."*Microwave-Assisted Synthesis of Reduced Graphene Oxide/SnO₂ Nanocomposite for Oxygen Reduction Reaction in Microbial Fuel Cells*" ACS Appl. Mater. Interfaces 8 (2016) 4633.

[19] S. Bag; B. Mondal, A. K. Das, C. Retna Daj. *Nitrogen and Sulfur Dual-Doped Reduced Graphene Oxide: Synergistic Effect of Dopants Towards Oxygen Reduction Reaction*" Electrochimica Acta 163 (2015) 16.

[25] G. Periyasamy, K. Annamalai; I. M. Patil; B. Kakade. "*Sulfur and nitrogen co-doped rGO sheets as efficient electrocatalyst for oxygen reduction reaction in alkaline medium*" Diamond & Related

Materials

[26] Y. Su; Y. Zhang; X. Zhuang; S. Li; D. Wu; F. Zhang; X. Feng. "*Low-temperature synthesis of nitrogen/sulfur co-doped three-dimensional graphene frameworks as efficient metal-free electrocatalyst for oxygen reduction reaction*" Carbon 62 (2013) 296.

[28] K. Qu, Y. Zheng; S. Dai; S. Z. Quiao "*Graphene oxide-polydopamine derived N, S-codoped carbon nanosheets as superior bifunctional electrocatalysts for oxygen reduction and evolution*" Nano Energy 19 (2016) 373.

[39] J. Zhang; J. Wang; Z. Wu; S. Wang, Y, Wu; X. Liu. "*Heteroatom (Nitrogen/Sulfur)-Doped Graphene as an Efficient Electrocatalyst for Oxygen Reduction and Evolution Reactions*" Catalysts 8 (2018) 475.

[48] B. P. Vinayan, Z. Zhao-Karger; T. Diemant; V. S. K. Chakravadhanula; N. I. Schwarzburger; M. A. Cambaz; R. J. Behm; C. Kübel; M. Fichtner. «*Performance study of magnesium–sulfur battery using a graphene based sulfur composite cathode electrode and a non-nucleophilic Mg electrolyte*» Nanoscale 8 (2016) 3296.

[52]H. Zhang; X. Liu; G. He; X. Zhang; S. Bao; W. Hu. "*Bioinspired synthesis of nitrogen/sulfur co-doped graphene as an efficient electrocatalyst for oxygen reduction reaction*" Journal of Power Sources 279 (2015) 252.

114 (2021) 108338.

[59] F. Pan; Y. Duan; X. Zhang; J. Zhang "*A Facile Synthesis of Nitrogen/Sulfur Co-Doped Graphene for the Oxygen Reduction Reaction*" ChemCatChem 8 (2016) 163.
